# Practical Treatment of Pyorrhea Alveolaris

**Published:** 1905-08-15

**Authors:** Austin F. James

**Affiliations:** Chicago


### PRACTICAL TREATMENT OF PYORRHEA ALVEOLARIS.

BY AUSTIN F. JAMES. D.D.S., CHICAGO.

From my clinical experience, I am forced to conclude that pyorrhea is a local disease, and cured by local treatment. While many writers upon the subject advance the theory that pyorrhea is caused by systemic conditions and that it is a constitutional disease, my experience has been that whatever the cause may be, it can be controlled and cured by local treatment, where there has not been a too great loss of the alveolar processes.

I find that in nine out of ten mouths which come under my observation, I can find local conditions which will cause pyorrhea; in some part of the mouth we will find a loosened irritated condition of the gum margins and an accumulation of food in a fermentive stage under these margins and upon the surface of the teeth—the teeth being more or less sensitive to the touch. With properly shaped instruments we will find a ridge or nodule of calcareous deposits upon the neck of the tooth.

While we may find these conditions in many parts of the mouth it does not necessarily mean that the patient is suffering with pyorrhea, but it is this condition, if allowed to remain, which will cause absorption of the alveolar processes and formation of pockets where more food and moisture w.ll accumulate, and the subsequent formation of pus, and a generally infected condition of the mouth.

The first step in the treatment of pyorrhea must be the prophylactic treatment, so far as the mouth in general is concerned. Then the removal of deposits, and here we come to the most difficult thing that a dentist has to do—th one operation which requires the greatest skill in the handling of instruments and a greater development of the senses than any other operation the dentist is called upon to perform. And with all this, a conscience that will not allow him to believe that he has done his work well until he has made one more effort to prove it.

After having given especial thought and effort on the treatment of this disease for a period of more than eight years, I can make the positive statement that I am curing many of these cases by surgical treatment; that is, the selection of properly shaped instruments, suitable for getting at every surface of each tooth, and knowing that I have thoroughly removed all the deposits of calculi and carefully smoothed and polished the necks of the teeth.

For the removal of these deposits I have selected the most suitable instruments from the many sets found in the dental supply houses, my choice being the Younger set improved by Dr. Good, the Mawhinney and some of Dr. D. D. Smith's set, all of which I here exhibit. For polishing I make use of the orange wood and finely powdered pumice.

The removal of deposits can be accomplished without the use of anesthetics and with no pain to the patient if care is used in selecting the proper instrument for each position.

The patient is then instructed as to how to properly care for the gums and teeth, and in the use of brushes for massageing the gums and cleansing the teeth.

When pus pockets are found after removing deposits and they have been syringed with warm water, a 20 percent solution of argyrol is used, feely injecting the solution into the pockets. The argyrol is a thorough non-irritating disinfectant and invariably prevents soreness following the surgical treatment, and is the only drug I find necessary in the treatment of pyorrhea aside from a good mouth wash for continuous use.

Patients are told to spend from three to five minutes twice a day massaging the gums with brushes, thinking what they are doing and timing the treatment to the full time by the clock.

Patients do not always respond to the simple telling, and as this is the only preventive we have against a recurrence of the disease, I allow myself to become a crank upon this subject with pyorrhea patients, and talk massaging of the gums and care of the mouth from the time they enter my private office until they retire, and in this way have taught many of them to become enthusiasts and find that recurrence can be prevented as well as the teeth be made practically immune from decay.

The operator should never admit to himself that the deposits have been removed until the condition of the gums proves that it is so by their becoming firm and hard around the necks of the teeth.

When teeth are found to be so extremely sensitive that it is impossible to use instruments, this sensitiveness can be overcome by thoroughly polishing the teeth with orange wood and pumice, thereby mechanically removing the fermentive accumulation of food and secretions, and having the patients use neutralizing agents until a second sitting can be had, when instruments can be used without causing pain.

All instruments used should be the kind known as draw instruments, having rounded surfaces where they come in contact with the gum tissue; by the careful use of these instruments you will often be rewarded by the pleasant statement coming from patients that they have suffered no pain at all, after sittings of an hour or more duration.

In all cases where the teeth are loosened, retaining appliances must be used to put the parts at rest during the healing and reformation of tissues.

In many cases it is advisable to make permanent appliances, cementing them in place, as there is no possibility of the reformation of the alveolar processes, and the most we can hope to do is to destroy the pus pockets and keep them clean until filled in with new soft tissues.

While I do not claim to give anything new to the profession, this paper is meant to plead for more thorough work along this line and to insist that many more teeth can be saved than at the present time, and at least we can prevent pyorrhea alveolaris.—*Review.*

